# Supplementary material for: Dual Mkk4 and Mkk7 Gene Deletion in Adult Mouse Causes an Impairment of Hippocampal Immature Granule Cells
Source: Int J Mol Sci. 2021 Sep 2;22(17):9545. doi: 10.3390/ijms22179545 (PMC8430506; doi:10.3390/ijms22179545)
Supplement: Supplementary file 1 [file ijms-22-09545-s001.zip › ijms-1324323-supplementary.pdf]

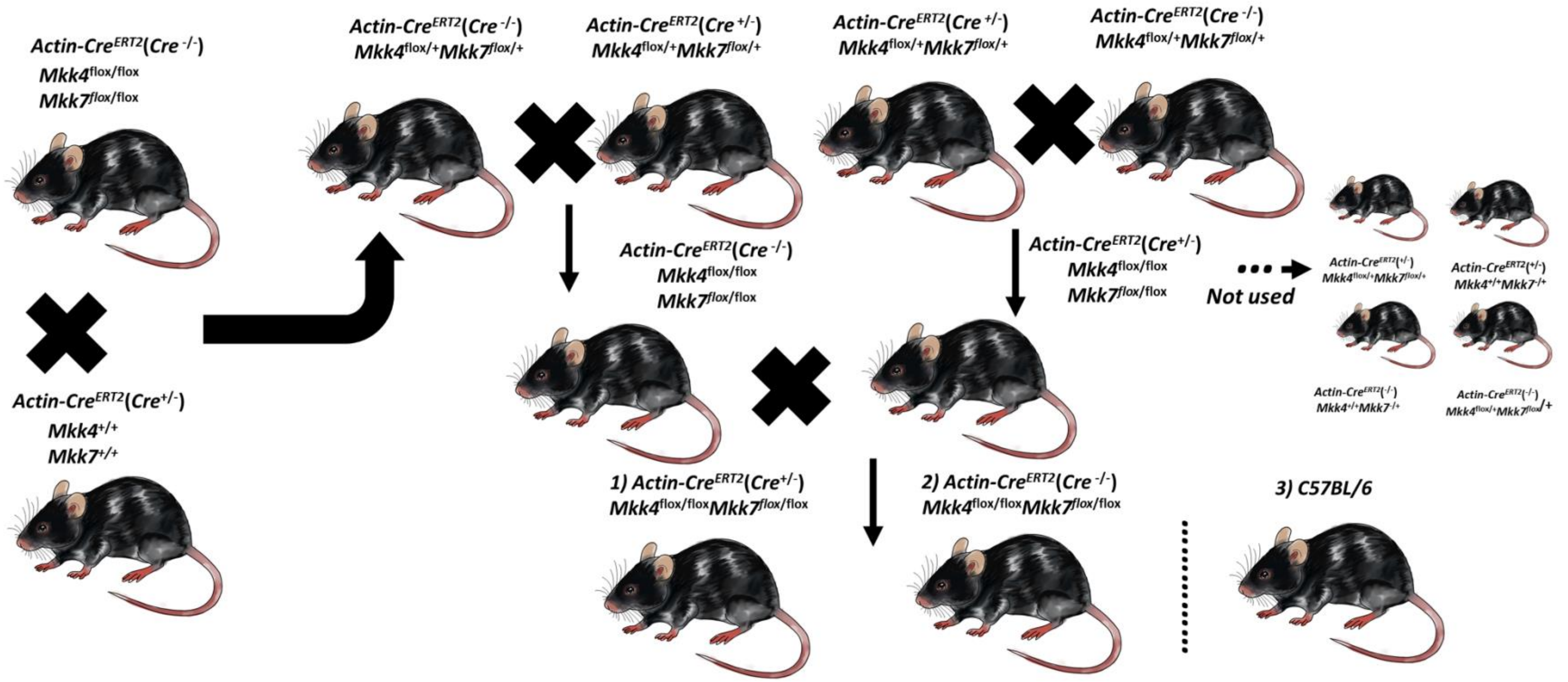

Figure S1. Generation of *Actin-Cre*^{ERT2}(*Cre*$^{+/-}$),*Mkk4*$^{flox/flox}$,*Mkk* $^{flox/flox}$ mice. Crossbreeding employed to get mice with the genotype handled in this investigation. Hemizygous *Actin-Cre*^{ERT2}(*Cre*$^{+/-}$), *Mkk4*$^{flox/flox}$,*Mkk7*$^{flox/flox}$ mice **(1)** was used to induce dual deletion of *Mkk4* and *Mkk7* genes after tamoxifen administration *Actin-Cre*^{ERT2} (*Cre*$^{-/-}$), *Mkk4*$^{flox/flox}$,*Mkk7*$^{flox/flox}$ **(2)** and wild-type C57BL/6 **(3)** mice were used in control groups.

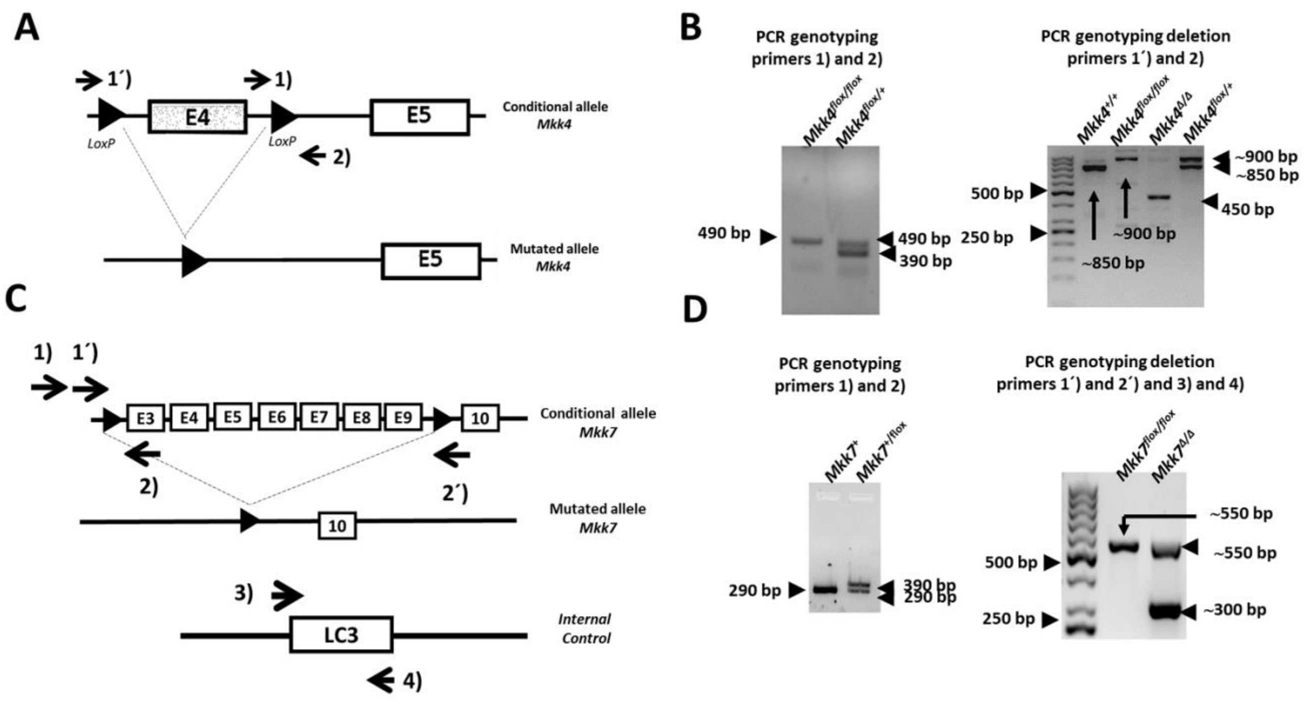

Figure S2. Strategy to genotype and to detect the deletion of *Mkk4* and *Mkk7* genes. A. Genomic region of conditional allele *Mkk4*, LoxP sequences flanked exon 4. Primers used for genotyping are 1) and 2). Primers employed for detecting deletion are 1´) and 2). **B.** In the left side, representative image of agarose gel for PCR genotyping test shows the band for *Mkk4*$^{+}$(390 bp) and *Mkk4*^{flox}(490 bp). On the right side, an image of agarose gel for PCR deletion test shows a band for *Mkk4*$^{+}$ (850 bp), *Mkk4*^{flox} (900 bp) and *Mkk4*$^{\Delta}$ (450 bp). **C.** Genomic region of conditional allele *Mkk7*, LoxP sequences flanked exon 3 to exon 9. Primers used for genotyping are 1) and 2). Primers used for deletion are 1´) and 2´) plus a pair of primers 3) and 4) that amplifies a LC3 region, used as an internal control. **D.** Left, representative image of agarose gel for PCR genotyping test showing the band for *Mkk4*$^{+}$ (290 bp) and *Mkk4*^{flox} (390 bp). The right agarose gel shows bands obtained for PCR deletion, *Mkk7*$^{\Delta/\Delta}$ genotype shows a band of 300 bp (*Mkk7*$^{\Delta}$) for deletion and 550 bp for Internal Control, *Mkk7*$^{flox/flox}$ genotype shows only a band of 550 bp (Internal Control).
